# Nutritional, Physiochemical, and Biological Value of Muffins Enriched with Edible Insects Flour

**DOI:** 10.3390/antiox10071122

**Published:** 2021-07-14

**Authors:** Ewelina Zielińska, Urszula Pankiewicz, Monika Sujka

**Affiliations:** Department of Analysis and Evaluation of Food Quality, University of Life Sciences in Lublin, Skromna Str. 8, 20-704 Lublin, Poland; ewelina.zielinska@up.lublin.pl (E.Z.); monika.sujka@up.lublin.pl (M.S.)

**Keywords:** edible insects, entomophagy, biological activity, muffins with insects, mealworm, cricket

## Abstract

Edible insects are gaining attention as a novel food; however, studies with their use in food are still limited. This study aimed to determine the chemical composition, physical parameters, sensory acceptance, and biological properties of muffins enriched with different levels of cricket (*Gryllodes sigillatus*) and mealworm (*Tenebrio molitor*) flours. The approximate composition was analyzed, along with the physical and textural properties, color, and consumer acceptance. Moreover, the antioxidant properties, starch digestibility, and glycemic index were determined in vitro. As we expected, the protein content in muffins supplemented with insect flour increased, while the carbohydrates content decreased. Moreover, the total phenolic content and antioxidant capacity against ABTS·^+^ and DPPH· increased correspondingly as the percentage of insect flour in the muffins increased. The estimated glycemic index was lower for the fortified muffins than the control (*p* < 0.05). Additionally, enriched muffins were accepted by consumers, and their taste positively surprised respondents (*p* < 0.05). Therefore, the results obtained are satisfactory as regards the use of insects for the supplementation of traditional products, and further research into the addition of insects to other nutrient matrices is needed. Furthermore, examining the effect of insect addition on in vivo food biological activity is highly desirable.

## 1. Introduction

One of the popular trends in food production concerns food fortification, which is related to the growing interest of consumers in this type of food. Food fortification is performed due to several factors, including improving the nutritional value of food, correcting deficiencies in nutrients or minerals, or enhancing health-promoting properties [1]. As a result, products with high protein, vitamin, amino acids, and mineral contents are gaining popularity [2]. Furthermore, not all food products are equally suitable for supplementation. For food fortification to be successful, supplemented foods should be consumed widely and in adequate quantities. For this reason, bakery products such as biscuits or bread, and also pasta, are very often chosen as a food matrix. This type of food can be fortified by the direct addition of a given ingredient during processing. This type of fortification results not only in an increase in the nutritional value of the product, but also changes its rheological and sensory characteristics [1]. 

Improving the nutritional quality of these types of foods is often achieved by the addition of selected food groups such as legumes, oilseeds, or herbs [1]. However, the trend in food fortification is changing to the use of unconventional sources of nutrients and bioactive ingredients. This is caused, for example, by the need to transform the food industry, leading to rational food management, the use of new technologies, and alternative raw materials, as well as waste management, with a reduced negative impact on the environment. In this context, the use of edible insects as food ingredients can be a beneficial alternative to enriching conventional foods [3]. Insects are undoubtedly an important source of protein, but also of micronutrients and bioactive compounds [4,5,6].

Insects are a natural source of substances with biological activity, including antibacterial, antiviral, and anti-inflammatory activities [7]. In traditional medicine, some species of edible insects are used to treat inflammatory diseases [8,9]. Stull et al. found that eating a breakfast enriched with 25 g of cricket powder for 2 weeks reduced circulating levels of the pro-inflammatory cytokine TNF-α in the plasma of 20 healthy adults [10]. In turn, in myoblast cell lines, the cricket *Brachytrupes orientalis* has been shown to reduce intracellular production of reactive oxygen species (ROS), lipid peroxidation, and increase the expression of Nrf2 and glutathione S-transferase proteins, involved in the redox response to stress, after stimulation with high glucose levels [11]. Moreover, hydrolysate and peptide fractions from insects such as *Tenebrio molitor*, *Schistocerca gregaria*, and *Gryllodes sigillatus* exhibited the ability to inhibit enzyme activity, such as lipoxygenase and cyclooxygenase-2, as well as displaying free radical scavenging activity in vitro [12,13]. These insect species also inhibited enzymes involved in the pathogenesis of metabolic syndrome in vitro [14]. However, the major interest is in the antioxidant properties of flours derived from insects [15,16], because antioxidants are among the most studied issues in both food science and nutrition [17]. Di Mattia et al. showed that water-soluble extracts of grasshoppers, silkworm, and crickets display five-fold higher values of antioxidant capacity, measured as TEACaq (mmol TE/100 g), than fresh orange juice [18]. There are many in vivo studies indicating the key role of antioxidant activity in the treatment of many diseases, such as cardiovascular disorders, diabetes, cancer, and neurodegenerative disorders. Furthermore, antibacterial, antiviral, anti-inflammatory, and anticancer activities are often associated with antioxidant properties [17,19,20]. It should be noted, however, that most studies focus on in vitro analysis, which can only provide an overview and direction for further research including clinical trials. It should therefore be noted that the results obtained from in vitro tests can only indicate the potential properties of the tested raw materials; nevertheless, they are necessary to carry out as a first step in the overall assessment of the bioactivity of the product.

Furthermore, the use of insects in the food industry has other positive benefits related to food sustainability. Insects have a high dry matter conversion of ingested food, which reduces breeding costs [21,22]. Moreover, insect farming requires less feed and water, with a reduced environmental impact, than conventional protein sources [23,24]. Although food sustainability is becoming increasingly important, consumer acceptance of insect consumption is still limited [25,26]. Although entomophagy is common in many countries around the world, mainly in Southeast Asia and South America, there is a significant barrier in terms of attitudes towards the use of insects as food, especially in Western societies, which show low acceptance of entomophagy [27]. The acceptance and popularity of entomophagy vary considerably across countries, as the willingness to consume depends on the cultural region, as confirmed by a survey of German and Chinese consumers. This cross-cultural comparison was made based on consumers’ willingness to consume various insect-based products; processed (e.g., cricket flour-based cookies) and unprocessed (e.g., crickets). Chinese consumers rated all insect-based foods more positively in terms of taste, nutritional value, familiarity, and social acceptance, and had a greater willingness to consume tested foods than did German consumers. Moreover, no differences were observed between the ratings of processed and unprocessed foods incorporating insect powders by the Chinese consumers, while German consumers had a greater willingness to eat processed insect-based foods [28]. For the introduction of insects as a food in the West, it is important to design suitable products to gain appropriate consumer acceptance, because the simple addition of insects to known products is not sufficient; monitoring of the physicochemical, as well as sensory characteristics of such products, is required [29]. 

Recently, there has been a lot of work focusing on designing products with insects and studying their properties. The most commonly fortified products are bakery products [3,30], bread [2,31,32,33], biscuits [34,35,36], snacks [37,38], pasta [39], tortillas [40], and meat products such as pâtés [41,42], frankfurters [43], sausages [44], or burgers [45,46]. This research aimed to study the incorporation of flours from two different insects (mealworms *Tenebrio molitor* and crickets *Gryllodes sigillatus*) into wheat muffins, by assessment of physical parameters, nutrient composition, bioactive properties, and sensory characteristics.

## 2. Materials and Methods

### 2.1. Materials

Wheat flour (type 500) (protein 10%, fat 1%, carbohydrates 76%), milk (protein 3%, fat 3.2%, carbohydrates 4.7%), rapeseed oil, eggs (protein 7.3%, fat 5.7%, carbohydrates 0.3%), sugar, and baking powder were purchased from a local market. The mealworms *Tenebrio molitor* (Linnaeus, Coleoptera: Tenebrionidae) (larvae), and crickets *Gryllodes sigillatus* (Fabricius, Orthoptera: Gryllidae) (adult) were obtained from a local breeder, Bugstore (Krakow, Poland). Insects were fasted for two days to empty their digestive tract and next they were frozen for 48 h at −20 °C and lyophilized. Afterward, the insects were ground in a laboratory grinder (IKA A11 basic), and the obtained flour was passed through a 20 mesh sieve. All the chemicals and reagents used were of analytical grade.

### 2.2. Muffin Preparation

Muffins were prepared in four variants, differing in insect flour addition (2%, 6%, 10% (*w*/*w*) by replacing an equivalent amount of wheat flour) or with the addition of whole insects (6% (*w*/*w*) of wheat flour) for each of the studied insect species. Control muffins were prepared without the addition of insects. The recipe of the muffins contained ingredients commonly used for their preparation, and these were wheat flour (43.4%), milk (31.2%), rapeseed oil (11.6%), eggs (8.1%), sugar (5.4%), and baking powder (0.3%). Wet and dry ingredients were combined separately and then combined briefly to blend the ingredients. The batter (60 g) was poured into a muffin cup and baked at 190 °C for 20 min in a preheated oven. The biscuits were then allowed to cool at room temperature and subjected to a consumer acceptance analysis, as well as color measurements and texture profile analysis, which were carried out within 24 h of baking. Until then, the muffins were stored in airtight containers and next were frozen at −20 °C so that further analyses could be performed.

### 2.3. Nutrient Composition 

The samples of flours and muffins were estimated for their moisture, ash, fat, and protein content by employing the standard methods of analysis [47]. Carbohydrates were determined by difference, by the following formula: 100—(weight in grams (protein + fat + ash + moisture) in 100 g). The conversion method was used for the determination of the nutritional value [48].

### 2.4. Color Measurements

An EnviSense colorimeter NH310 (EnviSense, Lublin, Poland) was used to measure the color of the muffins. Color differences were recorded in the CIE L*a*b* scale, in terms of lightness (L*) and color (a*—redness, b*—yellowness). Chroma (C*) and hue (h°) were also recorded. Furthermore, the total color difference (ΔE) was calculated using the following formula:

(1) $$\Delta E\;=\sqrt{{\Delta L}^{*2}+{\Delta a}^{*2}+{\Delta b}^{*2}},$$

where ΔL*, Δa*, and Δb* are differences in the L*, a*, b* values between the reference sample and the test sample, respectively.

### 2.5. Textural Analysis of Muffins

The texture profile analysis (TPA) of the obtained muffins was performed one day after baking, according to the method described by Mildner-Szkudlarz et al. [49], with a slight modification. A TA.XTplus texture analyzer (Stable Micro System Co. Ltd., Vienna Court, UK) equipped with a 5 kg load cell and a cylindrical plunger probe of 10-cm diameter was used. The measurement was performed at the pre-test speed: 2.0 mm/s, and test speed: 2.0 mm/s. The muffins were cut into 25 mm thick slices (the top and bottom layers were discarded) and they were compressed twice to 50% of their original height. During the test, hardness, springiness, cohesiveness, chewiness (product of hardness × cohesiveness × springiness, N), and resilience were measured. Texture analysis of muffin samples was carried out in triplicates for each sample.

### 2.6. Consumer Acceptance Analysis

The organoleptic analysis was carried out on freshly baked (1 h after baking) muffins. The evaluation of the muffins was performed by 26 panelists aged from 20 to 29 years, who were randomly selected among the students of the University of Life Sciences in Lublin, Poland. The color, aroma, texture, taste, and overall acceptability of the muffins were assessed on a scale of 1 to 5 (5—excellent, 1—extremely unsatisfactory). The conditions of the evaluation were constant. 

### 2.7. Consumer Study

Consumer study was carried out in two parts—before and after the organoleptic evaluation of muffins. The test consisted of a set of statements scored on a 5-point Likert scale ranging from “strongly disagree” to “strongly agree” [50]. This study aimed to record consumers’ opinions about the muffins before and after tasting them and to learn about their willingness to further consume insects, considering the perceptions they had in the sensory analysis. The statements assessed before the sensory evaluation of the muffins were as follows:

(1) I am curious about the taste of muffins
(2) All muffin variants look equally appetizing
(3) The addition of insect flour made me reluctant to taste them
(4) The addition of whole insects made me reluctant to taste them
(5) Adding insect flour to muffins is a better idea than adding whole insects
(6) The addition of whole insects to muffins causes more reluctance to taste them than the addition of insect flour.

In turn, after sensory analysis of the muffins, consumers were asked to assess the following items:

(1) The taste of muffins with insect/insect flour surprised me positively
(2) I would like to try other insect products
(3) Muffins with insect flour are acceptable to me, I would eat them in the future
(4) Muffins with whole insects are acceptable to me, I would eat them in the future
(5) I would recommend that others try baked products with insect/insect flour.

### 2.8. Antioxidant Properties

#### 2.8.1. Extraction of Bioactive Compounds

The crushed in mortar muffin samples (1 g) were shaken with 10 mL of 4:1 ethanol/water (*v*/*v*) in a laboratory shaker. After 120 min, the samples were centrifuged at 3000× *g* for 10 min. The supernatant was stored at −20 °C for further analysis [51]. 

#### 2.8.2. DPPH Radical Scavenging Activity

The Brand-Williams, Cuvelier, and Berset [52] method with slight modification was used to evaluate the DPPH^•^ scavenging activity. A mixture containing 0.1 mL volume of the sample and 0.9 mL of a 6 µM solution of DPPH^•^ in 75% methanol was prepared. The absorbance of this mixture was read at 515 nm after 30 min of the reaction, with 75% methanol used as a blank. The scavenging effect was calculated using the equation:

Scavenging activity (%) = [1 − (A sample/A control)] × 100,(2)

where A sample is the absorbance of the mixture of sample and DPPH^•^; A control is the absorbance of the control (DPPH^•^ solution).

The results were expressed as Trolox equivalent antioxidant activity (TEAC) values (mM Trolox).

#### 2.8.3. ABTS Radical Scavenging Activity

The method of Re et al. [53], with slight modifications, was used to evaluate the ABTS^•+^ scavenging activity. The radical solution was prepared with ABTS and potassium persulfate, diluted in water at 2.45 mM concentration. The solution was kept in the dark for 16 h to develop a radical and then was diluted to reach an absorbance value around 0.7 at 734 nm. Then, 0.05 mL of each sample was added to 2.95 mL of the ABTS^•+^ solution. After 30 min of the reaction the absorbance of the mixture was read at 734 nm. Distilled water was used as a blank. The scavenging effect was calculated according to the equation:

Scavenging activity (%) = [1 − (A sample/A control)] × 100,(3)

where A sample is the absorbance of the mixture of sample and ABTS^•+^; A control is the absorbance of the control (ABTS^•+^ solution).

The results were expressed as Trolox equivalent antioxidant activity (TEAC) values (mM Trolox).

#### 2.8.4. Total Phenolic Content

The TPC of different samples was determined according to the Folin–Ciocalteu spectrophotometric method [54]. Briefly, 0.04 mL of extracts was diluted with 3.16 mL of distilled water, and 0.2 mL of Folin–Ciocalteu’s reagent. After vigorous stirring in a vortex mixer, 0.6 mL of saturated sodium carbonate was added and incubation was carried out at 40 °C for 30 min. The absorbance was measured at 725 nm. A control solution was prepared accordingly, but without extract addition, replacing it with water. The results were expressed as mg of gallic acid equivalents (GAE) per gram of sample.

### 2.9. In Vitro Digestibility of Muffins

The procedure of Monro et al. [55], with slight modifications, was used to determine in vitro digestibility of the starch. Briefly, 30 mL of water and 0.8 mL of 1 M HCl were added to 1 g of the sample to reach a pH of 2.5. To start the gastric digestion, 1 mL of 10% pepsin solution in 0.05 M HCl was added and samples were incubated for 30 min at 37 °C with stirring (130 rpm). In the next step, the small intestine phase was started by the addition of 2 mL of 1 M NaHCO_3_ and 5 mL of 0.1 M phosphate buffer (pH 6), and next 4.6 mg amyloglucosidase and 5 mL of 2.5% pancreatin in 0.1 M phosphate buffer (pH 6). The volume of hydrolysates was made up with distilled water to 55 mL. Digesta aliquots of 1.0 mL were removed at 20, 30, 60, 90, 120, and 180 min from the start of amylolysis, and then added to 4 mL absolute ethanol in a tube and mixed to inactivate the enzymes.

### 2.10. Rapidly and Slowly Digested Starch Contents

The procedure of Soong, Tan, Leong, and Henry [56] was used to measure the reducing sugars released during in vitro digestion of the muffins, and on this basis the rapidly digestible starch (RDS) and slowly digestible starch (SDS) levels were evaluated. A dinitrosalicylic acid (DNS) colorimetric method was used to determine sugars as monosaccharides released during in vitro digestion. Briefly, 50 mL of supernatant was added to 0.25 mL of acetate buffer containing 0.4% invertase and 1% amyloglucosidase and incubated at room temperature for 30 min to complete the depolymerization into monosaccharides. Next, 0.75 mL of DNS mixture (0.5 mg/mL glucose, 4 M NaOH, and DNS reagent at a 1:1:5 ratio) was added and heated for 15 min at 95–100 °C. After cooling, samples were diluted with 4 mL distilled water and the absorbance was read at 530 nm against the blank. 

The amount of sugars released was presented as the mg of glucose/g of each sample and mg of glucose/g dry weight of the sample. The amount of rapidly digestible starch (RDS) was assessed as the amount of reducing sugars determined in the sample after 20 min from the start of intestinal digestion, whereas the amount of slowly digestible starch (SDS) was calculated as the difference between the amount of reducing sugars measured after 120 min of intestinal digestion and RDS content.

### 2.11. Glycemic Index In Vitro

The method of Reis and Abu-Ghannam [57] was adapted to evaluate the in vitro glycemic index (GI) of the muffins. The digestion procedure described above was used in this procedure. Digesta aliquots of 1 mL were taken at 20, 30, 60, 90, 120, and 180 min after the start of intestinal digestion in the tubes, with 4 mL of absolute ethanol used to deactivate the enzymes. The GOPOD method was used for determination of the glucose content in the samples (mg glucose/g sample). Glucose content was plotted as a function of time, and the areas under the hydrolysis curves (AUC) were calculated. The ratio between the AUC of the sample and the AUC for the reference food (white bread) was used to calculate the hydrolysis index (HI) values expressed as percentages, which were normalized for the total carbohydrate available in each sample and reference. The glycemic index was predicted according to the equation defined by Goñi, Garcia-Alonso, and Saura-Calixto [58]:

GI (%) = 39.71 + 0.549 × HI,(4)

### 2.12. Statistical Analysis

All assays were performed in triplicate and the obtained data are presented as means ± SEM (the standard error of the mean). Statistical analyses were carried out using Statistica (version 13.0, StatSoft, Krakow, Poland) for comparison of means using ANOVA with post hoc Tukey’s honestly significant difference (HSD) test at the significance level *p* < 0.05. A one-way ANOVA test was used in the questionnaire analysis as well. The insect flour addition and TPC were compared with the values obtained for antioxidant capacity (ABTS and DPPH) using Pearson’s correlation test.

## 3. Results and Discussion

### 3.1. Nutrient Composition

Edible insects are known as a valuable source of protein, and the protein contents for the studied insects flours were determined as 54.29% for mealworm and 71.15% for cricket (Table 1). Therefore, the supplementation of muffins with insect flour resulted in the expected proportional increase in the protein content of muffins as the addition of flour increased. The protein content increased by 1.3%, 17.1%, and 21.8% for muffins supplemented with 2%, 6%, and 10% of mealworm flour, respectively, and 16.3% and 34% for muffins supplemented with 6% and 10% of cricket flour, respectively, compared to reference muffin, in fresh muffin weight. However, considering the difference in muffin moisture, an increase in protein content occurred in all supplemented muffins, successively by 7.8%, 18.7% and 29.1% for muffins supplemented with 2%, 6%, and 10% of mealworm flour, respectively, and 8.3%, 20%, and 37.4% for muffins supplemented with 6% and 10% of cricket flour, respectively, compared to the reference muffin. The higher protein content of cricket flour resulted in a higher increase in protein content in muffins supplemented with it compared to mealworm flour. Moreover, an increase in the protein content of the muffins resulted in a decrease in the carbohydrate content (*p* < 0.05). The fat content of the tested flours was significantly different; 28.01% in mealworm flour and 14.92% in cricket flour, which is reflected in the fat content of the supplemented muffins. The studied muffins, except for samples with 2% insect flour added, had a statistically significantly higher fat content (*p* < 0.05) compared to the control, which was a consequence of the higher fat content of the insects flours than wheat flour. However, these differences were not large because the main source of fat in muffins is the oil added according to the recipe. In turn, the differences in energy value were mainly due to the different moisture contents of the muffins. All supplemented muffins had a higher moisture content than the control, which may have been due to the addition of an ingredient with a higher water absorption capacity, which influenced the properties of the bread [59]. Differences in the moisture content of muffins can also be related to the muffin baking process. Therefore, to eliminate the effect of moisture on the content of essential nutrients in the muffins, their content as % of dry weight is presented in Table 1.

### 3.2. Color Measurements

The results of the crumb color measurements of the muffins containing insect flours showed different brownish intensities (Table 2). Increasing the amount of insect flour decreased the lightness (L*) (except for samples with 2% flour addition, as also noted by other authors for muffins enriched with cricket flour [60]), redness (a*) for muffins fortified with 2% of insects’ flour and whole insects, and the yellowness (b*) of all samples, while increasing total color difference (∆E). Color is an important feature for baked products, because it is one of several factors influencing consumer acceptance [3]. Changing the color of bakery products to a darker color does not necessarily mean a lower consumer evaluation. Other authors suggested the similarity of these products to the color of whole grain products [60]. The color of the supplemented muffins was darker than the control, similarly to products enriched with insect flour a noted by other authors [3,33,35,39,60]. In turn, the dark color of fortified products may depend on several factors, such as the color of the raw materials; insect flour is darker than wheat flour. González et al. (2019) noted that the addition of *Hermetia illucens* flour caused a stronger darkening of the bread than the addition of mealworm *T. molitor* or cricket *A. domestica* flour. However, the addition of all flours caused browning of the crumb in a similar way to lentils and chickpea flours that resemble in color insect flours. Furthermore, the color changes which were observed could have been the result of the conversion of the nutrients present in the muffins, e.g., proteins from insects, through the heat treatment applied. Samples with more insect flour contained a higher amount of protein, which could intensify Maillard reactions [61]. Moreover, the fortified muffins had less saturated colors than the control (lower C* values). This means that the shade of the color changed from yellowish to redder.

The total color difference (∆E) between the control muffin and the supplemented muffins was significant because color differences >3 are obvious to the human eye [62]. A higher difference was noted for mealworm flour addition, ranging from 4.98 to 18.52, with a total color difference for muffins with cricket flour addition of 4.85–12.09.

### 3.3. Textural Analysis of Muffins

The texture parameters, including hardness, springiness, cohesiveness, chewiness, and resilience of the studied muffins, are presented in Table 3. The supplementation of muffins with insect flour in our experiment gave the muffins a softer texture compared to the control, as evidenced by the significant (*p* < 0.05) decrease in hardness, springiness, resilience, cohesiveness, and chewiness of the muffins. Generally, muffins enriched with insect flour showed a reduction of textural parameters values, with a few exceptions; the hardness and chewiness for muffins with the addition of 10% cricket flour remained constant (*p* < 0.05), and the cohesiveness for muffins with the addition of 6% and 10% cricket flour increased slightly in comparison to the control muffin. This indicates that the addition of cricket flour has a more significant effect on the texture of muffins than mealworm flour. 

The springiness value reflects the muffin’s ability to return to its original shape when the deforming force is removed, and this is associated with the freshness of a product, with a high-quality muffin having a higher springiness value. The control muffin was significantly (*p* < 0.05) springier (0.84%) than the fortified muffins for which the springiness decreased to the same extent (0.74–0.77%). Moisture content can also affect springiness; the supplemented muffins were characterized by a higher moisture than the control. Less springiness indicates a tendency to crumble. Moreover, a lower springiness and cohesiveness indicate a lower specific volume and less aerated structure in muffins [63]. This is an unfavorable change in terms of consumer acceptance. In turn, a lower chewiness indicates that it requires less time to chew a piece of the muffin before swallowing [3]. Consumers may feel difficulty during consuming muffins with very high chewiness, so this is a useful change. Moreover, reducing the hardness of muffins is another advantage that has a positive impact on consumer acceptance of the product. Other muffins fortified with millet, flaxseed, or fenugreek seed husk yielded similar results with a decrease in textural parameter values [64,65,66]. This is due to differences in the baking characteristics of wheat flour and the other flours used for enrichment muffins, as well as different macronutrient contents. It is also worth emphasizing the different moisture content of the selected muffins (Table 1). Although some studies suggest that a difference in moisture content of bakery goods (e.g., bread) of about 5% does not significantly affect the texture parameters [67], this factor should not be completely excluded as a significant variable affecting the texture of the tested product. Special steps should be taken when processing the dough to ensure that all muffins have the same moisture content, in commercial practice especially; and then the obtained texture parameters might be used to describe a scientific principle. Our study should be considered preliminary, and further research is needed to explain in-depth the effect of insect flour addition on the textural parameters of baked goods.

### 3.4. Consumer Acceptance Analysis and Consumer Study

The organoleptic analysis of muffins on a 5-point scale was carried out among students of the University of Life Sciences in Lublin, and the results are presented in Table 4. Generally, the control muffin received the highest scores for individual characteristics (color—4.38, consistency—4.23, smell—4.31, and taste—4.15), as well as for overall acceptability (4.38), and an increase in the contribution of insect flour in muffins resulted in a proportionally slight decrease in scores. However, it is important to emphasize that a statistical analysis of the results did not show significant differences at a 0.05 significance level. This allows the conclusion that the tested percentage additions of insect flour achieved organoleptic acceptance among the consumers.

In addition to the organoleptic evaluation, a survey of consumer opinions about the insects was conducted. The test consisted of a set of statements scored on a 5-point Likert scale ranging from “strongly disagree” to “strongly agree”. The consumer study was carried out in two parts; before and after the organoleptic evaluation of muffins to contrast the opinions. Generally, consumers were curious about the taste of muffins (3.77) (Table 5). However, the addition of whole insects to the muffins made consumers more reluctant to taste them than the addition of insect flour (4.62). Moreover, females supported this statement more strongly (4.82) than males (3.50) (*p* < 0.05). These results correspond well with our previous studies, where the desire to eat insects was expressed to a greater extent by the males (52.48%) than by the females (35.25%), as well as those obtained for German consumers by Orsi et al. [68]. De Boer et al. [69] considered that males are more inclined to take on challenges than females, which may have affected this result.

After the organoleptic evaluation of the muffins, the consumers were positively surprised about the taste of the muffins (3.84) (Table 6). Moreover, the more skeptical females were more surprised (4.04) than males (2.75) (*p* < 0.05). Muffins with insect flour were more acceptable (3.73) than those with whole insects (2.00). In addition, males were more open about these issues than females, as well as about the possibility of tasting other products with insects or recommending them to others. The results of the study confirm that neophobia is an important factor when proposing new products with insects. Similar observations were noted by Barton et al. (2020) [70], that if consumers have a positive experience with entomophagy, they are more willing to try insects in the future. Additionally, by integrating the crickets into a familiar product, the participants’ disgust decreased, so processing insects is a way to enhance their acceptance by consumers.

### 3.5. Antioxidant Properties

The antioxidant properties of muffins and insect flours were evaluated based on the determination of total phenolic content (TPC) and the ability to neutralize DPPH· and ABTS·^+^ (Table 7). The lowest TPC was determined in the control muffin (6.04 mg GAE/100 g) and the highest in the mealworm and cricket flours (351.05 and 515.76 mg GAE/100 g, respectively). Similar results were obtained by Di Mattia, Battista, Sacchetti, and Serafini [18] for extracts from *Acheta domesticus* cricket, 299 mg GAE/100 g, and mealworm (*T. molitor*), 406 mg GAE/100 g. All muffins enriched with insect flour had a higher TPC than the control muffin. Generally, the TPC increased with the higher percentage of insect flour content in muffins. Among muffins with the addition mealworm, the TPC ranged from 6.64 to 34.11 mg GAE/100 g, and for muffins with the addition of cricket from 17.53 to 112.06 mg GAE/100 g. The higher TPC in cricket enriched muffins was due to 1.5 times higher TPC in cricket flour than in mealworm flour.

A similar dependence was observed for the ability to neutralize DPPH· and ABTS·^+^. Insect flours showed the strongest antiradical effect, and the cricket flour had a stronger scavenging activity (DPPH· scavenging activity—2.273 mM TE/100 g, and for ABTS·^+^—1.15 mM TE/100 g) than the mealworm flour (DPPH·scavenging activity—1.21 mM TE/100 g, and for ABTS·^+^—0.93 mM TE/100 g). Similarly, Di Mattia et al. [18] reported TEAC values against ABTS·^+^ at 2.37 mM TE/100 g for *A. domesticus* and 0.89 mM TE/100 g for *T. molitor*.

These results correlated with the TPC, where the cricket flour also had the highest TPC of all the samples tested. Moreover, the antioxidant capacity against ABTS·^+^ and DPPH· increased correspondingly as the percentage of insect flour in the muffins increased; there is a strong positive correlation between insect flour addition, TCP content, and the ability to neutralize ABTS·^+^ and DPPH· (R = 0.970–0.999). Similarly, in our previous studies, the antioxidant activity levels of shortcake biscuits increased as the concentration of mealworm flour in the recipe increased [35]. This proves that the bioactive potential of the products is enhanced by the addition of insects.

Both our previous studies [12,25,71], as well as those of other authors [16,18,72], indicated that insects have strong antioxidant properties. The presence of antioxidants in our diet plays an important role in preventing oxidative stress-related diseases, the so-called civilization diseases such as cardiovascular disease or diabetes, as well as the degenerative process associated with aging [12]. Di Mattia et al. [18] noted that in vivo efficiency of foods rich in antioxidants is highly dependent on their bioavailability, which is a very important comment. Additionally, they note that a high antioxidant content in foods is an important requirement for testing the antioxidant potential of novel foods. Therefore, the determination of the in vitro antioxidant potential of insect-enriched products at high levels is a valuable observation for further studies leading to confirmation of this activity in vivo.

### 3.6. Rapidly and Slowly Digested Starch Contents and Glycemic Index In Vitro

Rapidly digestible starch (RDS) is defined as the portion of starch in a product that is digested within 20 min of food consumption and that causes a rapid rise in blood glucose levels. Slowly digestible starch (SDS) is starch that is completely digested in the small intestine, but at a slower rate than RDS, i.e., within 20–120 min after ingestion, helping to stabilize glucose metabolism [55]. The potential impact of muffins on glycemia could be strong, as evidenced by the significantly higher RDS content than SDS content (Table 8). However, the addition of insect flour to muffins resulted in a reduction of RDS and an increase in SDS content (*p* < 0.05). Generally, as the insect flour content in muffins increased, there was a proportional increase in SDS content, with a decrease in RDS content. More significant changes were noted with cricket flour supplementation than mealworm flour. The most significant changes were observed for the 10% addition of cricket flour; RDS content decreased from 254.17 mg/g of sample (338.04 mg/g d.w. sample) in the control to 162.18 mg/g (221.13 mg/g d.w. sample), while the SDS content increased from 26.45 mg/g (35.18 mg/g d.w. sample) in the control to 61.29 mg/g (83.57 mg/g d.w. sample). Similar results were obtained previously for shortcake biscuits supplemented with mealworm. Samples that contained the highest amounts of mealworm flour had significantly lower contents of RDS and higher contents of SDS than the control [35]. 

Muffins baked with rice, wheat, oat, corn, and barley flour showed similar SDS contents, but with a much higher RDS content, in the range of 387 to 445 mg glucose/g muffin [56]. The digestibility of starch present in foods, especially those subjected to different types of heat treatment, is dependent on other components present in the food. These other components may include proteins, and interactions between them are crucial modifications to starch digestibility [73]. In line with these reports, insect proteins may interact with starch, resulting in higher SDS content.

Moreover, we determined that an increase in the addition of cricket flour caused a decrease in RDS content, but in the case of mealworm the decrease in RDS content was observed only up to a certain point, of 6%, and further addition of flour did not affect the RDS content. The reason for this phenomenon may be the lower protein content of mealworms (54.29%) than crickets (71.15%) and the need for an even higher addition of flour to record successive decreases in RDS. Another reason may be the type and manner of interactions occurring between the starch and the components contained in the insects; the protein [74], fiber [75], fat [76], and phenolic compounds [77]. Furthermore, the addition of whole crickets caused a higher decrease in RDS than the addition of the same amount of cricket flour, which is probably related to the presence of these macronutrients in a different form. Non-starchy ingredients contained in food affect starch digestibility by forming ordered starch structures and/or reducing enzyme activity [78]. In general, starch interacted with lipids or proteins through electrostatic and hydrophobic interactions and this increased the ordered structures of starch to slow down the starch digestibility. These interactions play a key role in modulating digestibility and may occur under different conditions [79]. Protein affects the functional properties of starch, such as the rate of gelatinization and digestion of starch, because protein can form a continuous layer surrounding the starch granules [80,81]. Lipids also limit the availability and binding of enzymes to starch molecules. Lipids existing outside the starch granule can form complexes with amylose after heat treatment, limiting the swelling and solubility of starch granules [82]. Fiber is also one of the factors involved in starch digestion. The inclusion of soluble fibers increases the resistance of starch to enzymatic degradation because it can result in the formation of a viscous protein–fiber–starch network that can trap starch granules, limiting the release of glucose [75]. An example is the effect of guar gum in lowering postprandial glycemia and insulinemia by influencing intestinal physiology, and directly inhibiting the first step of biochemical starch degradation, i.e., amylolysis [83]. This action can occur in two ways: by altering the viscosity of the food matrix, affecting the rate of starch digestion; and can also play the role of a physical barrier, reducing enzyme-substrate interactions [84]. Therefore, the addition of insect meal as a source of ingredients that interact with starch promotes a reduction in starch digestibility through the complex transformations and reactions that occur during the heat treatment of muffins. Changes in starch digestibility can be manifested by both a decrease in RDS and an increase in SDS, as well as a lack in the availability of digestive enzymes to starch at the tested digestion time of 120 min. Foods containing carbohydrates can be classified according to their potential effect on postprandial blood glucose levels (‘glycaemic response’), which can be assessed as the glycaemic index (GI) [85]. The predicted glycemic index (pGI) was lower for the fortified muffins than the control (*p* < 0.05) (Table 8). A lower predicted glycemic index was observed for muffins with the highest SDS content; muffins with the addition of 10% of cricket flour, 6% of whole insects, and 10% of mealworm flour (pGI 79.3, 80.74, and 81.16, respectively), whereas the pGI of the control muffin was calculated as 92.22. These results suggest important low-glycemic characteristics of insect flours. Modulating the postprandial blood glucose response (by consuming foods with a low GI) has positive health effects, therefore further research is warranted in this area. Attempts are made to lower the glycemic index of products by adding various ingredients to the base recipe. Examples would be resistant starch, dextrins, or lentil flour, which decreased the pGI of muffins by 3.6 [86].

## 4. Conclusions

Edible insects can be incorporated into bakery products to improve their nutritional value, mainly in terms of protein content. The proposed food matrix was muffins, but this is just one example of presenting insects in a way that is attractive to the consumer. Based on the consumer evaluation, we can conclude that the addition of insects in the form of flour is a better ingredient than the addition of whole insects, as this provided a higher consumer acceptance.

The fortification of muffins with insect flours also affected their physical characteristics. Increasing the amount of insect flour decreased the lightness (L*), while increasing the total color difference (∆E). The supplementation of muffins with insect flour gave the muffins a softer texture compared to the control, as evidenced by the significant (*p* < 0.05) decrease in hardness, springiness, resilience, cohesiveness, and chewiness of the muffins.

In addition to studying the effect of insect flour addition on the physicochemical properties of muffins, the effect of their fortification on the potential biological activity of the products was also investigated. The antioxidant capacity against ABTS·^+^ and DPPH·, as well as total phenolic content (TPC), increased correspondingly as the percentage of insect flour in the muffins increased. The addition of insect flour to muffins resulted in a reduction of rapidly digested starch (RDS) and an increase in slowly digested starch (SDS) content (*p* < 0.05). Furthermore, this fortification resulted in a lower predicted glycemic index. This is important information because of the great interest in lower glycemic index products by people who often struggle with various conditions such as obesity, diabetes, and insulin resistance. Confirmation of the ability of insects to lower the glycemic index in vivo would allow them to be used to produce foods of this nature.

The results of these analyses indicate that there are many positive benefits to using insects in traditional foods. This encourages further research, in which it would be valuable to use other food matrices and extend the evaluation to a complete sensory analysis of the obtained products, which is a necessary step for marketing this type of product in Western countries.

## Figures and Tables

**Table 1:** Nutritional value of muffins.

Insect Species	Insect Flour Substitution	Protein (%)	Fat (%)	Ash (%)	Carbohydrates (%)	Moisture (%)	Energy Value (kJ/100 g)
**Control**	–	7.8 ± 0.55 ^bc^	15.32 ± 0.19 ^c^	0.9 ± 0.05 ^b^	51.17 ± 0.85 ^a^	24.81 ± 0.89 ^d^	1569 ± 8.0 ^a^
**Mealworm** ***(Tenebrio molitor)***	2%	7.9 ± 0.55 ^bc^	14.14 ± 0.38 ^de^	0.88 ± 0.07 ^b^	47.75 ± 0.67 ^b^	29.33 ± 0.03 ^b^	1469 ± 11.2 ^e^
	6%	9.13 ± 0.64 ^abc^	16.68 ± 0.13 ^a^	0.95 ± 0.05 ^b^	47.43 ± 0.55 ^bc^	25.86 ± 0.59 ^cd^	1579 ± 13.52 ^a^
	10%	9.5 ± 0.66 ^ab^	16.39 ± 0.1 ^ab^	0.98 ± 0.05 ^b^	44.0 ± 0.42 ^d^	29.13 ± 0.8 ^b^	1516 ± 10.2 ^cd^
	whole insects 6%	8.63 ± 0.6 ^bc^	15.51 ± 0.41 ^bc^	0.95 ± 0.05 ^b^	45.28 ± 0.64 ^de^	29.63 ± 0.89 ^b^	1490 ± 13.5 ^de^
	pure insect flour	54.29 ± 1.2	28.01 ± 0.18	1.39 ± 0.05	11.59 ± 0.05	4.72 ± 0.08	2157 ± 13.7
**Cricket** ***(Gryllodes sigillatus)***	2%	7.63 ± 0.53 ^c^	13.55 ± 0.52 ^e^	0.82 ± 0.08 ^b^	45.96 ± 0.72 ^cd^	32.04 ± 1.07 ^a^	1412 ± 12.22 ^f^
	6%	9.07 ± 0.64 ^abc^	15.17 ± 0.17 ^c^	0.93 ± 0.06 ^b^	47.74 ± 0.23 ^b^	27.09 ± 0.26 ^c^	1527 ± 15.56 ^bc^
	10%	10.45 ± 0.73 ^a^	16.42 ± 0.48 ^ab^	1.0 ± 0.09 ^b^	45.47 ± 0.58 ^de^	26.66 ± 0.46 ^c^	1558 ± 13.74.^ab^
	whole insects 6%	9.3 ± 0.65 ^abc^	14.82 ± 0.29 ^cd^	0.95 ± 0.06 ^b^	47.68 ± 0.34 ^b^	27.25 ± 0.8 ^c^	1517 ± 11.6 ^cd^
	pure insect flour	71.15 ± 1.1	14.92 ± 0.08	4.35 ± 0.08	4.83 ± 0.02	4.75 ± 0.09	1844 ± 10.3
		**% of dry weight**					
		**Protein**	**Fat**	**Ash**	**Carbohydrates**		
**Control**	–	10.37 ± 0.73 ^c^	20.38 ± 0.25 ^c^	1.19 ± 0.05 ^b^	68.06 ± 1.13 ^a^		
**Mealworm** ***(Tenebrio molitor)***	2%	11.18 ± 0.77 ^bc^	20.01 ± 0.54 ^c^	1.24 ± 0.07 ^b^	67.57 ± 0.95 ^ab^		
	6%	12.31 ± 0.85 ^abc^	22.49 ± 0.18 ^a^	1.28 ± 0.05 ^b^	63.92 ± 0.74 ^cde^		
	10%	13.39 ± 0.91 ^ab^	23.13 ± 0.14 ^a^	1.38 ± 0.05 ^b^	62.1 ± 0.59 ^de^		
	whole insects 6%	12.27 ± 0.84 ^abc^	22.04 ± 0.58 ^ab^	1.35 ± 0.05 ^b^	64.34 ± 0.91 ^cd^		
**Cricket** ***(Gryllodes sigillatus)***	2%	11.23 ± 0.77 ^bc^	19.93 ± 0.75 ^c^	1.21 ± 0.08 ^b^	67.63 ± 1.06 ^ab^		
	6%	12.44 ± 0.86 ^abc^	20.81 ± 0.23 ^bc^	1.28 ± 0.06 ^b^	65.47 ± 0.32 ^bc^		
	10%	14.25 ± 0.98 ^a^	22.39 ± 0.65 ^a^	1.36 ± 0.09 ^b^	62.0 ± 0.8 ^e^		
	whole insects 6%	12.79 ± 0.87 ^abc^	20.36 ± 0.4 ^c^	1.30 ± 0.06 ^b^	65.55 ± 0.47 ^bc^		

Values with different letters in the same column indicate significant differences (*p* < 0.05).

**Table 2:** Color determinants of muffins.

Insect Added to Muffin	Insect Flour Substitution	L*	a*	b*	C*	h°	∆E
**Control**	–	62.9 ± 0.61 ^b^	2.30 ± 0.15 ^a^	21.47 ± 0.45 ^a^	21.59 ± 0.46 ^a^	83.88 ± 0.35 ^b^	–
**Mealworm** ***(Tenebrio molitor)***	2%	66.42 ± 0.46 ^a^	1.49 ± 0.15 ^c^	18.05 ± 0.49 ^c^	18.11 ± 0.49 ^c^	85.29 ± 0.36 ^b^	4.98 ^g^
	6%	52.65 ± 1.41 ^d^	2.22 ± 0.09 ^a^	16.06 ± 0.55 ^e^	16.41 ± 0.56 ^f^	78.10 ± 0.08 ^d^	11.63 ^c^
	10%	46.12 ± 0.45 ^e^	3.38 ± 0.09 ^a^	13.62 ± 0.14 ^f^	13.80 ± 0.15 ^g^	80.74 ± 0.3 ^cd^	18.52 ^a^
	6% of whole insects	54.99 ± 0.9 ^d^	0.73 ± 0.07 ^d^	18.15 ± 1.26 ^c^	17.49 ± 0.62 ^d^	88.41 ± 0.66 ^a^	8.76 ^e^
**Cricket** ***(Gryllodes*** ***Sigillatus)***	2%	63.58 ± 0.94 ^ab^	2.02 ± 0.19 ^a^	16.67 ± 0.62 ^d^	16.80 ± 0.63 ^e^	83.12 ± 0.44 ^bc^	4.85 ^g^
	6%	59.26 ± 0.43 ^c^	3.51 ± 0.17 ^a^	13.16 ± 0.54 ^g^	13.62 ± 0.56 ^g^	75.08 ± 0.36 ^e^	9.15 ^d^
	10%	54.76 ± 0.56 ^d^	3.62 ± 0.16 ^a^	12.63 ± 0.23 ^h^	13.14 ± 0.24 ^h^	74.02 ± 0.6 ^e^	12.09 ^b^
	6% of whole insects	54.63 ± 1.32 ^d^	1.49 ± 0.08 ^c^	20.1 ± 0.05 ^b^	20.16 ± 0.05 ^b^	85.75 ± 0.23 ^ab^	8.42 ^f^

Values followed by a similar superscript in a column do not differ significantly (*p* < 0.05). Lightness (L*) and color (a*—redness, b*—yellowness). Chroma (C*) and hue (h°).

**Table 3:** Textural properties of muffins.

Insect Added to Muffin	Insect Flour Substitution	Hardness (N)	Springiness (%)	Cohesiveness	Chewiness (N)	Resilience
**Control**	–	121.4 ± 9.0 ^a^	0.84 ± 0.01 ^a^	0.42 ± 0.02 ^abc^	42.66 ± 4.5 ^a^	0.19 ± 0.01 ^a^
**Mealworm** ***(Tenebrio molitor)***	2%	91.2 ± 7.37 ^bc^	0.77 ± 0.02 ^b^	0.40 ± 0.04 ^c^	27.76 ± 2.0 ^cd^	0.15 ± 0.01 ^cde^
	6%	102.9 ± 5.5 ^ab^	0.76 ± 0.01 ^b^	0.37 ± 0.01 ^c^	29.06 ± 1.6 ^cd^	0.14 ± 0.01 ^de^
	10%	107.5 ± 9.3 ^ab^	0.76 ± 0.02 ^b^	0.44 ± 0.03 ^abc^	36.21 ± 2.9 ^abc^	0.17 ± 0.01 ^abc^
	6% of whole insects	124.2 ± 9.82 ^a^	0.77 ± 0.04 ^b^	0.41 ± 0.04 ^bc^	38.84 ± 0.77 ^ab^	0.18 ± 0.01 ^ab^
**Cricket** ***(Gryllodes sigillatus)***	2%	98.3 ± 15.0 ^ab^	0.75 ± 0.01 ^b^	0.40 ± 0.01 ^c^	29.90 ± 4.6 ^bcd^	0.13 ± 0.01 ^e^
	6%	67.7 ± 8.6 ^c^	0.75 ± 0.02 ^b^	0.48 ± 0.03 ^ab^	24.55 ± 3.5 ^d^	0.15 ± 0.01 ^cde^
	10%	121.4 ± 12.7 ^a^	0.74 ± 0.01 ^b^	0.49 ± 0.01 ^a^	44.40 ± 4.8 ^a^	0.16 ± 0.01 ^bcd^
	6% of whole insects	92.3 ± 10.3 ^bc^	0.77 ± 0.03 ^b^	0.38 ± 0.2 ^c^	26.46 ± 2.7 ^d^	0.16 ± 0.01 ^bcd^

Values different letters in the same column indicate significant differences (*p* < 0.05).

**Table 4:** Consumer acceptance analysis of muffins.

Insect Added to Muffin	Insect Flour Substitution	Color	Consistency	Smell	Taste	Overall Acceptability
**Control**	–	4.38 ± 0.9 ^a^	4.23 ± 0.7 ^a^	4.31 ± 0.8 ^a^	4.15 ± 0.9 ^a^	4.38 ± 0.6 ^a^
**Mealworm** ***(Tenebrio molitor)***	2%	4.27 ± 0.7 ^a^	4.15 ± 0.8 ^a^	4.04 ± 1.0 ^a^	3.65 ± 1.0 ^a^	4.00 ± 0.9 ^a^
	6%	3.54 ± 0.9 ^a^	3.85 ± 0.8 ^a^	4.15 ± 1.0 ^a^	4.00 ± 1.0 ^a^	4.00 ± 0.9 ^a^
	10%	3.15 ± 0.8 ^a^	3.27 ± 0.8 ^a^	3.54 ± 1.0 ^a^	3.31 ± 1.0 ^a^	3.50 ± 0.8 ^a^
	6% of whole insects	3.69 ± 1.0 ^a^	3.23 ± 1.0 ^a^	3.65 ± 0.9 ^a^	2.77 ± 1.0 ^a^	3.12 ± 1.0 ^a^
**Cricket** ***(Gryllodes sigillatus)***	2%	3.73 ± 0.6 ^a^	3.77 ± 0.8 ^a^	3.81 ± 1.0 ^a^	3.73 ± 0.6 ^a^	3.77 ± 0.7 ^a^
	6%	3.77 ± 1.0 ^a^	3.73 ± 1.0 ^a^	3.88 ± 1.0 ^a^	3.58 ± 1.0 ^a^	3.88 ± 1.0 ^a^
	10%	3.04 ± 1.0 ^a^	3.42 ± 0.9 ^a^	3.77 ± 0.9 ^a^	3.15 ± 1.0 ^a^	3.58 ± 0.8 ^a^
	6% of whole insects	3.58 ± 0.8 ^a^	3.50 ± 1.0 ^a^	3.73 ± 1.0 ^a^	3.19 ± 1.0 ^a^	3.23 ± 0.9 ^a^

Values with different letters in the same column indicate significant differences (*p* < 0.05).

**Table 5:** Results of statements before the sensory evaluation of the muffins (1 = strongly disagree; 5 = strongly agree) on the total sample and by gender.

Statement	Mean (sd)	DF	*t*-Value	*p*-Value	Male Mean (sd)	Female Mean (sd)	F-Value	*p*
I am curious about the taste of muffins	3.77(1.39)	1	−0.353	0.726	4.00(0.71)	3.72(0.30)	0.125	0.726
All muffin variants look equally appetizing	2.58(1.24)	1	0.566	0.566	2.25(0.63)	2.64(0.27)	0.320	0.577
The addition of insect flour made me reluctant to taste them	2.77(1.14)	1	1.500	0.147	2.00(0.56)	2.91(0.24)	2.251	0.147
The addition of whole insects made me reluctant to taste them	4.38(1.13)	1	1.688	0.104	3.50(0.54)	4.50(0.23)	2.850	0.104
Adding insect flour to muffins is a better idea than adding whole insects	4.38(0.85)	1	1.677	0.107	3.75(0.41)	4.50(0.18)	2.812	0.107
The addition of whole insects to muffins causes more reluctance to taste them than the addition of insect flour	4.62(0.90)	1	3.144	0.004	3.50(0.39)	4.82(0.16)	9.889	0.004 *

DF—Degree of Freedom; Signif. codes: * *p* < 0.05, Tukey test.

**Table 6:** Results of statements after the sensory evaluation of the muffins (1 = strongly disagree; 5 = strongly agree) on the total sample and by gender.

Statement	Mean (sd)	DF	*t*-Value	*p*-Value	Male Mean (sd)	Female Mean (sd)	F-Value	*p*
The taste of muffins with insect/insect flour surprised me positively	3.84(0.88)	1	3.154	0.004	2.75(0.96)	4.04(0.72)	9.947	0.004 *
I would like to try other insect products	3.23(1.30)	1	−1.298	0.206	4.00(1.41)	3.09(1.27)	1.686	0.206
Muffins with insect flour are acceptable to me, I would eat them in the future	3.73(1.25)	1	−1.359	0.187	4.50(0.58)	3.59(1.31)	1.848	0.187
Muffins with whole insects are acceptable to me, I would eat them in the future	2.00(1.23)	1	−1.344	0.192	2.75(1.71)	1.86(1.13)	1.806	0.192
I would recommend that others try baked products with insect/insect flour	3.42(1.17)	1	−1.073	0.294	4.00(0.81)	3.32(1.21)	1.152	0.294

DF—Degree of Freedom; Signif. codes: * *p* < 0.05, Tukey test.

**Table 7:** Antioxidant properties of muffins.

Insect Species	Insect Flour Substitution	TPC (mg GAE/100 g)	DPPH (mM TE/100 g)	ABTS (mM TE/100 g)
**Control**	–	6.04 ± 0.04 ^g^	0.04 ± 0.008 ^g^	0.27 ± 0.018 ^e^
**Mealworm** ***(Tenebrio molitor)***	2%	6.64 ± 0.1 ^g^	0.107 ± 0.02 ^f^	0.29 ± 0.023 ^de^
	6%	25.25 ± 0.18 ^e^	0.184 ± 0.01 ^de^	0.31 ± 0.01 ^cde^
	10%	34.11 ± 0.21 ^d^	0.223 ± 0.02 ^c^	0.37 ± 0.03 ^c^
	6% of whole insects	17.83 ± 0.15 ^f^	0.1 ± 0.004 ^f^	0.3 ± 0.03 ^de^
	insect flour	351.05 ± 3.55	1.21 ± 0.03	0.93 ± 0.03
**Cricket** ***(Gryllodes sigillatus)***	2%	17.53 ± 0.2 ^f^	0.159 ± 0.01 ^e^	0.35 ± 0.03 ^cd^
	6%	75.93 ± 0.52 ^b^	0.616 ± 0.01 ^b^	0.49 ± 0.02 ^b^
	10%	112.06 ± 1.12 ^a^	0.729 ± 0.02 ^a^	0.59 ± 0.03 ^a^
	6% of whole insects	66.73 ± 0.9 ^c^	0.192 ± 0.01 ^d^	0.31 ± 0.01 ^cde^
	insect flour	515.76 ± 5.2	2.273 ± 0.12	1.15 ± 0.0

Values with different letters in the same column indicate significant differences (*p* < 0.05).

**Table 8:** Rapidly and slowly digestible starch contents and in vitro glycemic index values for muffins.

Insect Species	Insect Flour Substitution	RDS (mg Glucose/g d.w. of Sample)	RDS (mg Glucose/g Sample)	SDS (mg Glucose/g d.w. of Sample)	SDS (mg Glucose/g Sample)	pGI
**Control**	–	338.04 ± 6.47 ^ab^	254.17 ± 4.86 ^a^	35.18 ± 1.49 ^f^	26.45 ± 1.12 ^f^	92.22 ± 0.42 ^a^
**Mealworm** ***(Tenebrio molitor)***	2%	344.62 ± 8.07 ^a^	243.54 ± 5.7 ^ab^	14.63 ± 1.36 ^h^	10.34 ± 0.96 ^h^	90.55 ± 0.38 ^ab^
	6%	321.8 ± 8.75 ^bc^	238.58 ± 6.49 ^bc^	45.97 ± 1.55 ^e^	32.58 ± 1.15 ^e^	84.50 ± 0.44 ^c^
	10%	321.8 ± 9.88 ^bc^	227.88 ± 7.0 ^cd^	78.21 ± 2.47 ^b^	55.43 ± 1.75 ^b^	81.16 ± 0.52 ^de^
	6% of whole insects	317.7 ± 5.97 ^cd^	223.58 ± 4.2 ^cde^	54.51 ± 2.02 ^d^	38.36 ± 1.42 ^d^	83.73 ± 0.36 ^cd^
**Cricket** ***(Gryllodes sigillatus)***	2%	312.7 ± 6.06 ^cd^	212.51 ± 4.12 ^e^	25.87 ± 0.79 ^g^	17.58 ± 0.54 ^g^	88.78 ± 0.42 ^b^
	6%	300.51 ± 5.34 ^d^	219.10 ± 3.89 ^de^	63.3 ± 2.54 ^c^	46.21 ± 1.85 ^c^	85.71 ± 0.44 ^c^
	10%	221.13 ± 4.77 ^f^	162.18 ± 3.5 ^g^	83.57 ± 1.64 ^a^	61.29 ± 1.2 ^a^	79.30 ± 0.39 ^e^
	6% of whole insects	249.21 ± 5.02 ^e^	181.30 ± 3.65 ^f^	60.01 ± 2.12 ^c^	43.66 ± 1.54 ^c^	80.74 ± 0.48 ^e^

Values with different letters in the same column indicate significant differences (*p* < 0.05).

## Data Availability

The data presented in this study are available on request from the corresponding author. The data are not publicly available due to their large amount.
